# Effect of Different Levels of Pressure Relieving Air-Mattress Firmness on Cough Strength

**DOI:** 10.1371/journal.pone.0146714

**Published:** 2016-01-07

**Authors:** Norimichi Kamikawa, Shunsuke Taito, Makoto Takahashi, Kiyokazu Sekikawa, Hironobu Hamada

**Affiliations:** 1 Department of Physical Analysis and Therapeutic Sciences, Graduate School of Biomedical and Health Sciences, Hiroshima University, Hiroshima, Japan; 2 Hiroshima College of Rehabilitation, Hiroshima, Japan; 3 Department of Clinical Support, Hiroshima University Hospital, Hiroshima, Japan; Harvard Medical School, UNITED STATES

## Abstract

Cough is an important host-defense mechanism. The elderly and patients who are severely ill cannot cough effectively when lying in the supine position. Furthermore, pressure relieving air-mattresses are recommended for preventing the development of pressure ulcers. In this study, we clarified whether or not the cough peak flow (CPF), an index of cough strength, is affected by different firmness levels of a pressure relieving air-mattress in healthy volunteers in the supine position. Fifty-two healthy young men participated. All the measurements were carried out on each participant in the supine position on a pressure relieving air-mattress. The participants were assessed at two firmness levels, a “hard” and “soft” mode. The CPF, forced vital capacity (FVC), maximal expiratory pressure (PEmax), and maximal inspiratory pressure (PImax) were determined for each mode. The sinking distance of the body into the mattress was measured without any activity and the difference between the sinking distances of the two firmness levels was determined. The CPF, FVC, PEmax, and PImax were determined for each mode. The sinking distance of the body into the mattress was measured and the difference between the sinking distances of the two firmness levels was determined. The CPF, FVC, PEmax and PImax values of the participants coughing on the mattress were significantly lower when the mattress was in “soft” than in “hard” mode. The differences between the sinking distances of the mattress in “soft” and “hard” modes were larger for the anterior superior iliac spine. A harder mattress may lead to increased CPF in healthy young men lying in the supine position, and increased CPF may be important for host defense.

## Introduction

Cough is an important host-defense mechanism, because it clears foreign material and secretions from the airway and prevents aspiration of food and fluid [1–3]. A voluntary cough consists of three phases, inspiratory, compressive, and expiratory [2]. A cough cannot be effective if even one of these phases is defective [4].

The cough peak flow (CPF) is an index of voluntary cough strength [5]. CPF values are affected by several factors, including lung volume and respiratory muscle strength, [4, 6–12] thorax expansion which is correlated with lung volume, [13, 14] and posture [15, 16]. In addition, a study of the effect of different body positions on CPF found that values were significantly higher for the standing position than for the supine position. Therefore, the standing position is encouraged for patients needing to cough to clear respiratory secretions [15]. However, elderly individuals and seriously ill patients who lie in the supine position may not be able to change their body position and produce an effective cough. Furthermore, pressure relieving mattresses have been recommended for preventing the development of pressure ulcers; [17, 18] and the interface pressure of the mattress has been usually set on low for elderly individuals or patients whose overall status has deteriorated [17, 19]. A pressure relieving air-mattress that is set on low leads to increased surface area of the body in contact with the mattress and decreased pressure on the skin in contact with the mattress. Therefore, clarifying whether or not pressure relieving air-mattress firmness levels affect the CPF of individuals in the supine position is important. To our knowledge, there have not been any published reports on the relationship between pressure relieving air-mattress firmness levels and the CPF.

In this study, we clarified whether or not the CPF of healthy volunteers in the supine position was affected by different firmness levels of a pressure relieving air-mattress.

## Materials and Methods

### Participants

The study participants consisted of 52 young, healthy male nonsmokers. Participants were excluded from enrollment in the study if they had apparent thorax and/or spine deformities or pulmonary disease. This study was approved by the ethics committee of the Hiroshima University Graduate School of Health Sciences (#1127). Written informed consent was obtained from all participants.

### Body position and pressure relieving air-mattress settings used for the assessments

All measurements were carried out with the participant in the supine position. Each participant was positioned on the pressure relieving air-mattress so that point where is his Jacoby line and medial plane intersected was at the center of the pressure relieving air-mattress. Both the upper and lower limbs were placed in the extended position, and both upper limbs were placed on each side of the body.

The pressure relieving air-mattress (GRANDE; Molten, Co., Ltd, Tokyo, Japan) consisted of a mattress with three independent layers, a pump, and an air hose that connected the mattress with the pump. The pressure relieving air-mattress conditions are manually selected by the operator: dehumidifier (off, weak, strong), firmness (hard, normal, soft, very soft), movement (none, pressure redistribution), and mattress thickness (thin 13cm, thick 18cm).

In the present study, the pressure relieving air-mattress firmness levels that were used for assessments were very soft or hard. “Very soft” and “hard” were defined as “soft” and “hard”, respectively. The firmness level was randomly selected for each participant in the experiments. The dehumidifier and movement setting were set at off, and the pressure relieving air-mattress thickness was set at thick (18cm) for every experiment.

### Measuring the inner pressure of the pressure relieving air-mattress

To confirm that the pressure relieving air-mattress was set at different levels of firmness, inner pressure levels were measured. A digital pressure gauge (PGI; Molten Co., Ltd, Hiroshima, Japan) was placed in the air inlet of the mattress, and the inner pressure levels were measured with the mattress in “soft” and “hard” mode for each participant lying on the mattress.

### Measurement of cough strength

CPF was measured using a peak flow meter (Assess, Philips Respironics, Tokyo, Japan). The examiner held the peak flow meter, and three CPF measurements were obtained after three practice coughs. The highest CPF value at each pressure relieving air-mattress firmness level was used for statistical analysis.

### Lung volume measurement

The forced vital capacity (FVC), an indicator of lung volume, was measured by a spirometer (Microspiro HI-701, Chest Co., Ltd, Tokyo, Japan) according to the standard methods of the American Thoracic Society [20]. The examiner held the sensor, and three FVC values were obtained. The highest FVC value at each pressure relieving air-mattress firmness level was used for statistical analysis.

### Measurement of respiratory muscle strength

The maximal expiratory pressure (PEmax), an index of the strength of the expiratory muscles, and the maximal inspiratory pressure (PImax), an index of the strength of the inspiratory muscles, were measured according to the method of Black and Hyatt [11] by a measurement device (AAM377, Minato Medical Science. Co., Ltd, Osaka, Japan) used to assess respiratory muscle strength, which was connected to an electronic spirometer (Autospiro AS-507, Minato Medical Science. Co., Ltd Osaka, Japan). The participants wore a nose clip, and the examiner held the device while measuring the pressures. For assessment of PEmax, each participant performed a maximal expiratory effort after a maximal inspiration. The PImax level was likewise assessed from a maximal inspiratory effort after a maximal expiration. The pressure was measured if it was maintained for at least 1.5 s. PEmax and PImax values were measured three times at each pressure relieving air-mattress firmness level after three practice attempts. The highest PEmax and PImax values at each pressure relieving air-mattress firmness level were used for statistical analysis.

### Measurement of body sinking distance

To quantify the amount the body sank into the pressure relieving air-mattress, the sinking distance was measured without any activity at three points on the body: the left lesser tubercle of the humerus, left anterior superior iliac spine (ASIS), and left patella. A marker was placed at each bony protuberance, and the distance from the edge of the bed to the marker was measured with the pressure relieving air-mattress in the “soft” and “hard” mode, by a three-dimensional motion analysis system (ICpro-2DdAF for Windows, Hu-Tech, Tokyo, Japan). The difference in the sinking distances of the “soft” and “hard” modes was calculated using the formula that follows.

Difference in sinking distance=sinking distance in“soft”mode - sinking distance in“hard”mode

### Statistical analysis

Results are expressed as means ± SD. One-way analysis of variance and the Tukey test were used to compare the differences in sinking distances measured at the three points on the body. The paired *t*-test was used to compare the inner mattress pressure, CPF, FVC, PEmax, and PImax values determined at the 2 different pressure relieving air-mattress firmness levels. Statistical analysis was performed using IBM SPSS ver. 21 software for Windows (IBM Japan, Tokyo, Japan). *P*-values less than 0.05 were considered statistically significant.

## Results

The characteristics and spirometric parameters of the participants were as follows: age 23.6 ± 5.3 years, height 171.5 ± 6.2 cm, weight 64.1 ± 9.7 kg, body mass index 21.8 ± 3.0 kg/m^2^, percent body fat 18.6 ± 5.5%, percent of predicted forced vital capacity 96.2 ± 15.9%, percent of predicted forced expiratory volume in 1 s 92.7 ± 16.2%.

Levels of the inner pressure of the pressure relieving air-mattress in the “soft” mode were significantly lower than pressure levels in the “hard” mode (soft 25.0 ± 1.9 hPa vs. hard 35.0 ± 1.9 hPa, *P* < 0.001). Table 1 shows the parameters related to CPF values at the 2 different pressure relieving air-mattress firmness levels. The values of all parameters for the “soft” mode were significantly lower than those for the “hard” mode.

**Table 1:** Cough parameters measured at two different pressure relieving air-mattress firmness levels.

	Soft	Hard	*P* value
CPF (L/min)	518.1 ± 118.4	567.5 ± 118.0	< 0.001
FVC (L)	4.59 ± 0.80	4.68 ± 0.88	< 0.001
PEmax (cm H_2_O)	86.1 ± 16.0	92.0 ± 17.6	< 0.001
PImax (cm H_2_O)	84.3 ± 16.2	88.6 ± 15.7	< 0.001

CPF, cough peak flow; FVC, forced vital capacity; PEmax, maximal expiratory pressure; PImax, maximal inspiratory pressure.

Values are expressed as mean ± SD.

**Fig 1:**
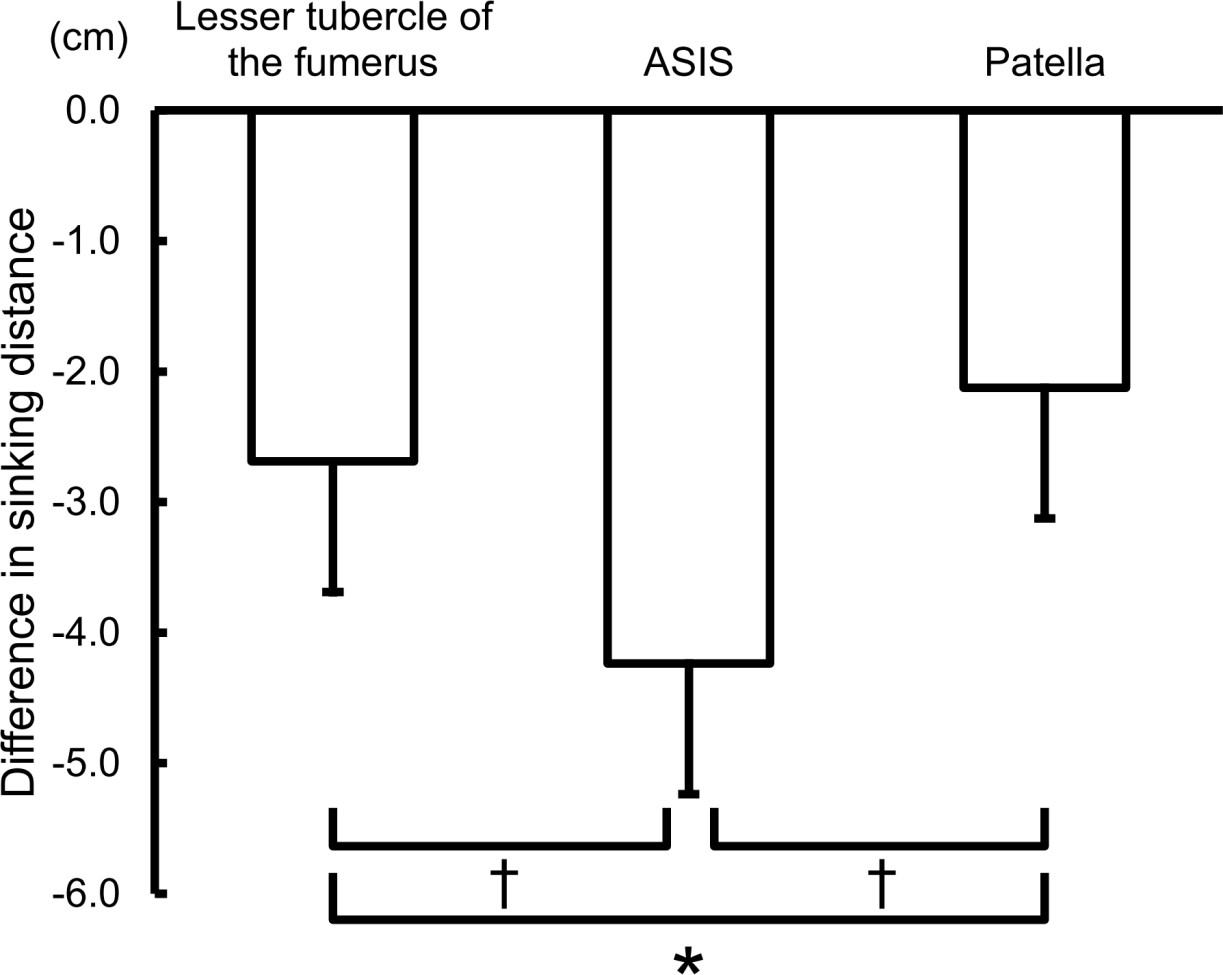
Differences in the sinking distances of three points between “soft” and “hard” mattress modes. ASIS, anterior superior iliac spine. *: *P* < 0.05; †: *P* < 0.001.

Fig 1 shows the differences in the sinking distances of the participants’ body between the pressure relieving air-mattress in “soft” and “hard” mode at three points on the body. The differences in the sinking distance at the left lesser tubercle of the humerus, left ASIS, and left patella were -2.7 ± 1.0 cm, -4.2 ± 1.4 cm, and -2.1 ± 0.8 cm, respectively. The differences in the sinking distances between the three points were statistically significant.

## Discussion

In this study, we studied whether pressure relieving air-mattress firmness levels affected the CPF of young healthy volunteers. The CPF, FVC, PEmax and PImax values of the participants with the pressure relieving air-mattress in the “soft” mode were significantly lower than the values with the pressure relieving air-mattress in the “hard” mode. These findings indicate that a harder mattress might be associated with a higher CPF, which is important for host defense.

To the best of our knowledge, this is the first study that demonstrated that cough strength was affected by different pressure relieving air-mattress firmness levels. To clarify the mechanisms involved in our CPF findings, we assessed several parameters associated with the production of cough with relation to the pressure relieving air-mattress firmness levels. The inspiratory phase of cough is affected by an increase in lung volume, which is produced by thoracic expansion and related to the strength of the inspiratory muscles [4, 14]. The compressive and expiratory phases of cough are affected by an increase in the intrathoracic pressure, as well as the strength of the expiratory muscles [2]. In this study, we measured the FVC, PImax, and PEmax, which are indices of lung volume, inspiratory muscle strength, and expiratory muscle strength, respectively.

The pressure relieving air-mattress firmness levels might change the normal curvature of the spine, which could affect the expansion of the thorax, lung volume, and degree of force exerted by the inspiratory and expiratory muscles. Yamamoto et al [21] reported that lower pressure levels of a pressure relieving air-mattress led to deeper immersion of the lumbar spine and buttocks with spinal curvature in healthy adults. Culham et al [22] reported that women with accentuated kyphosis of the spine demonstrated significantly lower vital capacity, lower total lung capacity, and decreased lateral expansion and vertical excursion of the ribs, compared to healthy women of the same age. Furthermore, two studies demonstrated that lung volume is correlated with both PImax and PEmax levels [23, 24]. In our study, the difference in the sinking distances of the ASIS measured at the “soft” and “hard” modes were larger than the differences for the lesser tubercle of the fumerus and patella. The FVC, PImax and PEmax values of the participants with the pressure relieving air-mattress in the “soft” mode were significantly lower compared to the values with the pressure relieving air-mattress in the “hard” mode. These results suggest that the deeper immersion of the lumbar spine and buttocks with accentuated spinal curvature of participants with the pressure relieving air-mattress in the “soft” mode might limit the expansion of the thorax, as reflected by the decreased FVC and PImax levels, and which resulted in lower PEmax.

The results of this study suggest that pressure relieving air-mattress firmness levels may affect the parameters associated with production of cough, including lung volume, inspiratory and expiratory muscle strength, and ultimately, the CPF.

## Study Limitations

The limitations of this study were as follows: first, our study participants were healthy young men; and second, we did not assess the details of the participants’ posture, such as spinal curvature. Studies that better delineate and assess such parameters as posture are needed for further evaluation of the effects of different pressure relieving air-mattress firmness levels on the cough strength of elderly individuals and patients. Such studies could provide information that aids in the selection of pressure relieving air-mattress firmness levels suitable for the elderly or severely ill patients, and suitable for pulmonary rehabilitation.

## Conclusions

This study of healthy young men in the supine position, who produced a voluntary cough, showed that the CPF, which is an index of cough strength, may be affected by different pressure relieving air-mattress firmness levels. The results suggest that a harder mattress may result in increased CPF values.

## References

[1] Fontana GA, Lavorini F. Cough motor mechanisms. Respir Physiol Neurobiol. 2006;152:266–81. 16600697 10.1016/j.resp.2006.02.016

[2] McCool F. Global physiology and pathophysiology of cough: ACCP evidence-based clinical practice guidelines. Chest. 2006;129:48–53. 10.1378/chest.129.1_suppl.48S 16428691

[3] Hadjikoutis S, Wiles CM, Eccles R. Cough in motor neuron disease: a review of mechanisms. QJM. 1999;92:487–94. 10627867 10.1093/qjmed/92.9.487

[4] Park JH, Kang SW, Lee SC, Choi WA, Kim DH. How respiratory muscle strength correlates with cough capacity in patients with respiratory muscle weakness. Yonsei Med J. 2010;51:392–7. doi: 10.3349/ymj.2010.51.3.392 20376892 10.3349/ymj.2010.51.3.392 PMC2852795

[5] Bach JR. The prevention of ventilatory failure due to inadequate pump function. Respir Care. 1997;42:403–13.

[6] Kera T, Maruyama H. Study of influence factor on maximal mouth pressure Part I. -Influence of posture-. J Phys Ther Sci. 2001;13:153–60.

[7] Kang SW, Bach JR. Maximum insufflation capacity. Chest. 2000;118:61–5. 10893360 10.1378/chest.118.1.61

[8] Polkey MI, Lyall RA, Green M, Leigh PN, Moxham J. Expiratory muscle function in amyotrophic lateral sclerosis. Am J Respir Crit Care Med. 1998;158:734–41. 9730998 10.1164/ajrccm.158.3.9710072

[9] Szeinberg A, Tabachnik E, Rashed N, McLaughlin FJ, Egland S, Bryan CA, et al Cough capacity in patients with muscular dystrophy. Chest. 1988;94:1232–5. 3191765 10.1378/chest.94.6.1232

[10] Kang SW, Kang YS, Sohn HS, Park JH, Moon JH. Respiratory muscle strength and cough capacity in patients with Duchenne muscular dystrophy. Yonsei Med J. 2006;47:184–90. 16642546 10.3349/ymj.2006.47.2.184 PMC2687626

[11] Black LF, Hyatt RE. Maximal respiratory pressures: Normal values and relationship to age and sex. Am Rev Respir Dis. 1969;99:696–702. 5772056 10.1164/arrd.1969.99.5.696

[12] Srour N, LeBlanc C, King J, McKim DA. Lung volume recruitment in multiple sclerosis. PLoS One. 2013;8:e56676 doi: 10.1371/journal.pone.0056676 23383293 10.1371/journal.pone.0056676 PMC3561294

[13] Fisher LR, Cawley MI, Holgate ST. Relation between chest expansion, pulmonary function, and exercise tolerance in patients with ankylosing spondylitis. Ann Rheum Dis. 1990;49:921–5. 2256739 10.1136/ard.49.11.921 PMC1004263

[14] Fink JB. Forced expiratory technique, directed cough, and autogenic drainage. Respir Care. 2007;52:1210–21; discussion 21–3. 17716387

[15] Badr C, Elkins MR, Ellis ER. The effect of body position on maximal expiratory pressure and flow. Aust J Physiother. 2002;48:95–102. 12047207 10.1016/s0004-9514(14)60203-8

[16] Lin F, Parthasarathy S, Taylor SJ, Pucci D, Hendrix RW, Makhsous M. Effect of different sitting postures on lung capacity, expiratory flow, and lumbar lordosis. Arch Phys Med Rehabil. 2006;87:504–9. 16571389 10.1016/j.apmr.2005.11.031

[17] Grey JE, Harding KG, Enoch S. Pressure ulcers. BMJ. 2006;332:472–5. 16497764 10.1136/bmj.332.7539.472 PMC1382548

[18] Cullum N, Deeks JJ, Fletcher AW, Sheldon TA, Song F. Preventing and treating pressure sores. Qual Health Care. 1995;4:289–97. 10156400 10.1136/qshc.4.4.289 PMC1055341

[19] Hofman A, Geelkerken RH, Wille J, Hamming JJ, Hermans J, Breslau PJ. Pressure sores and pressure-decreasing mattresses: controlled clinical trial. Lancet. 1994;343:568–71. 7906329 10.1016/s0140-6736(94)91521-0

[20] Miller MR, Hankinson J, Brusasco V, Burgos F, Casaburi R, Coates A, et al Standardisation of spirometry. Eur Respir J. 2005;26:319–38. 16055882 10.1183/09031936.05.00034805

[21] Yamamoto Y, Nakagami G, Mori T, Sakai K, Sanada H. Evaluation of preventive effect on buttocks immersion of independently controlled inner air cell pressure in air mattress. J Jpn Wound Ostomy Continence Manage. 2011;15:239–49.

[22] Culham EG, Jimenez HA, King CE. Thoracic kyphosis, rib mobility, and lung volumes in normal women and women with osteoporosis. Spine (Phila Pa 1976). 1994;19:1250–5. 8073317 10.1097/00007632-199405310-00010

[23] Troyer AD, Borenstein S, Cordier R. Analysis of lung volume restriction in patients with respiratory muscle weakness. Thorax. 1980;35:603–10. 7444828 10.1136/thx.35.8.603 PMC471343

[24] Nishimura Y, Hida W, Taguchi O, Sakurai M, Ichinose M, Inoue H, et al Respiratory muscle strength and gas exchange in neuromuscular diseases: comparison with chronic pulmonary emphysema and idiopathic pulmonary fibrosis. Tohoku J Exp Med. 1989;159:57–68. 2815076 10.1620/tjem.159.57

